# Anxiolytic and Antidepressant Effect of *Phaseolus vulgaris* on Animal Models

**DOI:** 10.1155/2024/5710969

**Published:** 2024-04-23

**Authors:** Rabia Munawwar, Sana Sarfaraz, Rahila Ikram, Talat Zehra, Humaira Anser, Huma Ali

**Affiliations:** ^1^Department of Pharmacology, Faculty of Pharmacy, Jinnah Sindh Medical University, Rafiqui H.J, Iqbal Shaheed Rd 75510, Karachi, Pakistan; ^2^Department of Pharmacology, Faculty of Pharmacy, University of Karachi, Main University Rd 75270, Karachi, NC-24, Deh Dih, Korangi Creek 74900, Karachi, Pakistan; ^3^Dean of Salim Habib University, Karachi, Pakistan; ^4^Department of Pathology, Faculty of Medicine, Jinnah Sindh Medical University, Rafiqui H.J, Iqbal Shaheed Rd 75510, Karachi, Pakistan; ^5^Principal of Institute of Pharmaceutical Sciences, Jinnah Sindh Medical University, Rafiqui H.J, Iqbal Shaheed Rd 75510, Karachi, Pakistan

## Abstract

An experimental study was conducted using rodents at different doses to evaluate the effect of *Phaseolus vulgaris* (red beans) on cage crossing, head dip, open field, elevated plus maze, and light and dark apparatus for anxiety and forced swim test for depression. The corticosterone level and histopathological evaluation was also done to correlate the antidepressive impact of the red beans. The study also identified the components responsible for the effect using GCMS. Based on the findings, red beans could be a potential non-pharmacological therapy for mild to moderate depressive patients. The anxiety model was conducted on mice weighing 20–25 gms. Group I was taken as control, group II as 500 mg/kg and group III as administered 1000 mg/kg. The tests were performed on 0th, 7th, 15th, 30th, 45th, and 60th day. The depression model research was conducted on albino rats weighing between 180 and 200 g, divided into four groups: a control group, a 500 mg/kg *Phaseolus vulgaris* group, a 1000 mg/kg *Phaseolus vulgaris* group, and a standard group treated with fluoxetine. The forced swimming test was performed on days 0, 7, 15, 30, 45, and 60, after which histopathological evaluations were conducted and blood samples were taken to assess corticosterone levels. GCMS was used to identify the constituents present in red beans, while optical spectroscopy was used to detect minerals and ions. Results showed that both doses of *Phaseolus vulgaris* possess anxiolytic effect and increased the struggling time of rats in depression model significantly, with the 1000 mg/kg dose showing more significant results than the 500 mg/kg dose. The GCMS results identified the presence of erucic acid, which causes an increase in *α*-amylase, thus reducing depression. Optical spectroscopy also showed that red beans contain zinc, which may increase BDNF and help in treating depression.

## 1. Introduction

Two major psychological problems are being faced by the world today, i.e., anxiety and depression. Anxiety in advanced form can be very dangerous. This behavioral condition may lead to several other health issues which are hazardous for humans. Discussions about anxiety have picked up, but this problem remains underexplored. This neurological problem develops into dangerous issues such as panic attacks, agoraphobia, generalized anxiety disorder, obsessive-compulsive disorder, and many other phobias [1]. Indications of anxiety are prevalent with a low differential ratio such as high blood pressure, overthinking, increase in heart rate, nervousness, shortness of breath, shivering, sweating, or sometimes diarrhoea [2]. Major depressive disorder (MDD) is a chronic medical condition that affects feelings, thinking processes, and actions. Sadness and a loss of interest in previously appreciated activities are symptoms of depression [3]. It can cause a wide range of mental and physical issues and reduce active participation at work and home. Depression affects about one in every 15 persons (6.7 percent). One out of every six persons (16.6%) will suffer from depression at some point in their lives [4]. Depression can strike at any age, but it is most common in late adolescence and early adulthood. Women are more prone to suffer from depression than males. According to a research study, one-third of women will have a significant depressive episode over their lives. Feelings of sadness and frustration that last for at least two weeks and are accompanied by other faulty symptoms will lead to depression [5].

Many studies have found that depression causes significant alterations in hippocampus neurons and amygdala hypertrophy [6]. Glial and neuron densities were lower in some brain areas, implying that severe depression may be linked to structural plasticity deficiencies [7]. Changes in corticosterone levels are monitored in severe depression [8]. These changes in the body may also trigger other pathological issues for humanity. Chronic depression can lead to abnormal function of glucagon and insulin, leading to diabetes [9]. Depression may also cause GIT (gastrointestinal tract) problems and also sometimes liver issues. One of the critical risk factors for depression is stressful life events [10].

Risk factors of depression in adolescents include increased substance abuse and suicides [11]. In women, depression may worsen the fertility rate, which ultimately causes more depression [12]. Depression increases the risk of anxiety and memory loss in the elderly and disrupts sleep and socializing [13].

Recent developments in neuroscience have improved our understanding of the pharmacological foundation of medical treatments for depressive disorders [14]. This new research could pave the way for better depression management. Dietary supplements demonstrate a slew of neurological advantages in humans [15]. These new studies are necessary for disease treatment and prevention. Healthy diets, indicated by high consumption of vegetables, fruit, fish, and whole grains, have been linked to more minor depressive symptoms and a lower risk of developing depressive symptoms in cross-sectional and prospective studies [16].

*Phaseolus vulgaris* is commonly known as French bean or kidney bean. They are the annual herbaceous legume that belongs to the genus *Phaseolus*, and they belong to the Fabaceae family. Kidney beans are rich in minerals (iron and zinc), proteins, and vitamins. It is a rich source of dietary fiber, which helps to reduce cholesterol absorption and eliminate fats from the body, so it is used for weight loss. It can decrease the average sugar level in the blood, so it is used as an antidiabetic agent. It has some role in kidney stones and urinary tract infections. Some other studies have shown that these beans greatly help in rheumatism [17]. Besides, it has some significant constituents to help develop and strengthen the neurons.

*Phaseolus vulgaris* contains isoleucines. The amino acid contents of the isoleucine are similar, except for threonine, lysine, and arginine [18]. Red beans also have phenols that act as antioxidants. The most abundant is catechin, which is 56 mg in 1 gm of sample [19]. Total amounts of carbohydrates present in *Phaseolus vulgaris* are 53.2 mg, proteins are 25.2 mg, and lipids are 2 mg [20]. These constituents are beneficial in treating many diseases and maintaining health.

The current study is conducted to evaluate the effects of red beans on depression, which may produce future benefits for humankind to deal with such neurological disorders. As the world is growing fast, there is a dire need to use natural products for the curing of mental issues.

## 2. Methodology

The anxiety model was prepared on mice of both sexes weighing 20–25 grams while the depression model was developed using albino male rats weighing between 180 and 200 grams. In the animal house of the Department of Pharmacology at the University of Karachi, rats were housed in their natural habitat of polypropylene rat cages. Rats were monitored closely to ensure that food and water and all environmental parameters such as temperature and humidity levels remained consistent at 25 ± 2°C and 50–60%, respectively. A 12-hour day and night cycle was also properly considered.

The guidelines laid out in Helsinki Resolution 1964 were followed when handling animals. The study was officially approved and allowed by the Institutional Board of Advanced Studies and Research by Resolution No. 31 (P).

### 2.1. Identification and Purchasing of Red Beans

The red beans are purchased from the local market and kept in room temperature in a zip-lock plastic bag. The seeds were identified with the number PVS-01-20 from the Department of Pharmacognosy, University of Karachi.

### 2.2. Preparation of *Phaseolus vulgaris* Powder

The beans were first selected to remove any contaminants and then washed and rinsed well with cold water. The beans were then cooked and dried until all water had evaporated. With an electric grinder, the dry beans were ground into powder form. With the help of a few drops of water, proper dose pellets were created.

### 2.3. Approval on Ethical Grounds

All authors have stated that all animal laboratory care guidelines were strictly followed. Animals were kept according to CCAC (Canadian Council on Animal Care) rules, which included providing standard feed and water to rats in a hygienic setting with a twelve-hour day and night cycle.

### 2.4. Model Design and Dose Schedule

Albino mice and albino rats were used in this investigation and were separated into four groups. Group I was designated as the control group and fed a standard diet. *Phaseolus vulgaris* 500 mg/kg was given to Group II. *Phaseolus vulgaris*, 1000 mg/kg, was given to Group III. Fluoxetine (5 mg/kg) was given to Group IV, which was considered standard. Pellets were supplied to each rat in the corresponding group daily for a period of 60 days. On day 0, day 7, day 15, day 30, day 45, and day 60, readings were taken on different apparatuses of both the models, respectively. On day 60, depression model rats were sacrificed and blood samples were drawn to evaluate the corticosterone levels. The hippocampus was also taken for the preparation of histology slides.

### 2.5. Anxiety Model Preparation

#### 2.5.1. Cage Crossing

The cage crossing method is used to evaluate anxiety models in rodents according to international standards. The equipment is built of plexiglass, which is square with a size of (26 × 26 × 26 cm) and is clear, allowing the rodent's activity to be observed easily. The floor is covered in sawdust. To avoid external disruption, the instrument is put in a quiet location. The animals were kept in cages because they felt at ease in their surroundings. The readings were recorded for a total of 5 minutes [21].

#### 2.5.2. Head Dip

The head dip test is a useful tool for determining how a medicine affects mouse behavior. The equipment is made out of a rectangular hardwood board that measures 35 cm height × 45 cm width and is enclosed. It has three 2.5 cm-diameter apertures on all sides. The rodent was placed in the apparatus' center and given 5 minutes to explore. The poking of the snout was seen [22].

#### 2.5.3. Open Field Test

In anxiety models, the open field test is often used to assess locomotor and behavioral activity. The equipment consists of a square container measuring 76 cm by 76 cm and measuring 42 cm in height. There are 25 squares on the floor. Each square has a 15 cm diameter. A central square of 15 × 15 cm can also be seen in the middle section. For a period of 10 minutes, the mouse was placed separately in the open fields and the number of central and peripheral crossings was noted [23].

#### 2.5.4. Light and Dark Test

The anxiety behavior of rodents is assessed using light and dark. The chamber is 44 × 21 × 21 cm in diameter and is made of propylene. It is divided into two halls by a tunnel (7.5 × 7.5 cm) that allows transit from one light compartment to the other dark compartment and vice versa. The translucent sides of the light chamber are exposed, and a lamp is installed to provide illumination. The dark compartment, which is sealed by a black cover, is painted black. The mice are placed in a light area for ten minutes to study the anxiolytic effect, and the time spent in the light environment is recorded. Rodents, on the other hand, like to reside in gloomy areas. The willingness of the mouse to explore the light-unprotected area demonstrates the anxiolytic effect, which is a characteristic that should be studied by the researcher [24].

#### 2.5.5. Elevated Plus Maze

One of the important tools for assessing anxiety is elevated plus maze. The apparatus has two open arms, two closed arms, and a center platform. These two type of arms are connected perpendicularly and the platform supports as a base which forms an elevated plus maze. Open arms that are 25 cm length × 5 cm width × 16 cm height each. Closed arms that are 25 cm length × 5 cm width × 16 cm height each with a center platform are utilized for the elevated plus maze test (5 × 5 × 0.5 cm). Closed arms have a high (16 cm) wall to surround the arm, whereas open arms have a very small (0.5 cm) wall to reduce the amount of falls. Plastic materials are used to construct the equipment. The platform is white with clear walls. The elevated plus maze apparatus comes in a variety of materials and colors [25].

### 2.6. Depression Model

#### 2.6.1. Force Swimming Apparatus

The forced swim test is a rat behavioral test used to analyze antidepressant medicines, novel compound antidepressant efficacy, and experimental treatments targeted at rendering or preventing depression-like conditions. Rats were placed in an impenetrable transparent tank filled with water, and their escape-related movement behavior was monitored. The forced swim test is simple to perform and requires little specialist equipment. The forced swim test can only be done successfully if certain procedural elements are followed and undue distress to the rodents is avoided. For the forced swim test, rodents were placed in a tiny, confined environment, such as a vast graduated cylinder halfway filled with water. Initially, the animal engages in a burst of activity in which it attempts to flee. The animal eventually stops moving and adopts peculiar immobility in which it only moves to keep its head above water. This physical immobility is regarded to be a sign of psychological distress [26].

The researchers measured the time between when the animal is placed in the chamber and when it becomes immobile and stops struggling. The time spent attempting to flee in rodent models of depression decreases, but antidepressants restore this decrease [27].

#### 2.6.2. Corticosterone Level Evaluation

To evaluate the corticosterone levels of rodents, the blood sample was drawn on the 60th day. The test tubes used to store blood sample were 3 ml Stat/Line green/yellow top, i.e., plasma separator tubes. First, prepurification of the blood sample was done, followed by the radioimmunoassay testing method [28].

#### 2.6.3. Histology of Hippocampus

On day 60, dissection of rodents was done to withdraw the brain of rats which were then sent to the laboratory for the gross examination of the hippocampus; the slides were then prepared to evaluate the neuronal changes in the hippocampus of depressed and treated rats.

#### 2.6.4. Gas Chromatography-Mass Spectroscopy

For GCMS, beans were crushed and dissolved in extracting solution which was methanol in which beans were soaked for a few hours and then placed in a sonicator for three hours to settle all the residues and then the supernatant solution was taken and placed in GCMS instrument for evaluation of fatty acids and flavonoids. The procedure is also known as ultrasonic extraction and analysis through GCMS in SIM (selected ion monitoring) mode [29].

#### 2.6.5. Inductively Coupled Plasma-Optical Emission Spectrometry

The technique is carried out for the detection of metal and ions in the apparatus known as inductively coupled plasma-optical emission spectrometry (ICP-OES). First, the beans were weighed at around 0.5 mg, then ashing was done for nine to ten hours, and then it was digested in acid. The nanoparticles were then injected into the nebulizer of the apparatus ICP-OES with the help of a technique known as the flow injection technique [30].

#### 2.6.6. Statistical Analysis for Anxiety Model

All the results of anxiety model were analyzed in SPSS 2 using one-way ANOVA followed by the Tukey test. A *p* value of less than 0.05 was considered as significant.

 
^*∗*^For control with other treated groups
 
^$^For comparison with red bean 500 mg

#### 2.6.7. Statistical Analysis for Depression Model

All the results of depression model were expressed in SPSS 26 using one-way ANOVA. The significance (*p* ≤ 0.005) of treated animals with red beans is compared with standard and control results.

- @ is used for the comparison with control.
- ? is used for the comparison with the standard.
- % is used for the comparison of 500 mg with 1000 mg.

## 3. Results

### 3.1. Anxiety Model

#### 3.1.1. Cage Crossing

**Figure 1:**
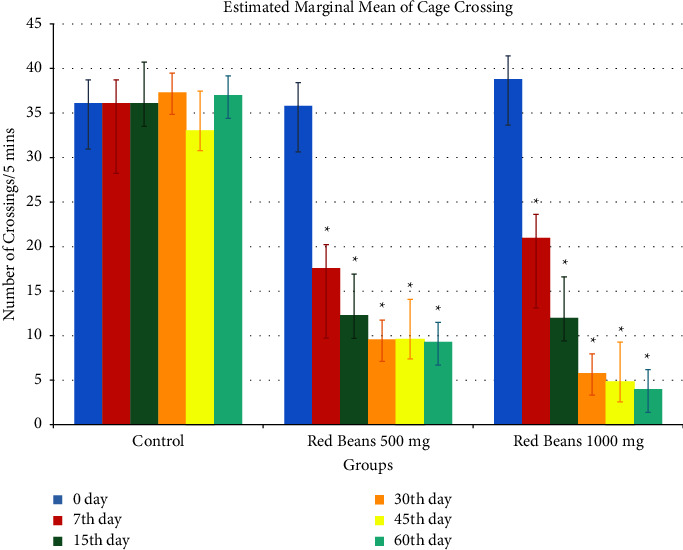
Cage crossing readings. ^*∗*^For control with other treated groups. ^$^For comparison with red bean 500 mg.

Figure 1 shows the effect of red beans of doses 500 mg/kg and 1000 mg/kg on cage crossing. Control group showed a significant difference with both the treated groups. 1000 mg/kg showed more anxiolytic effect on 60th day as compared to 500 mg/kg.

#### 3.1.2. Head Dip

**Figure 2:**
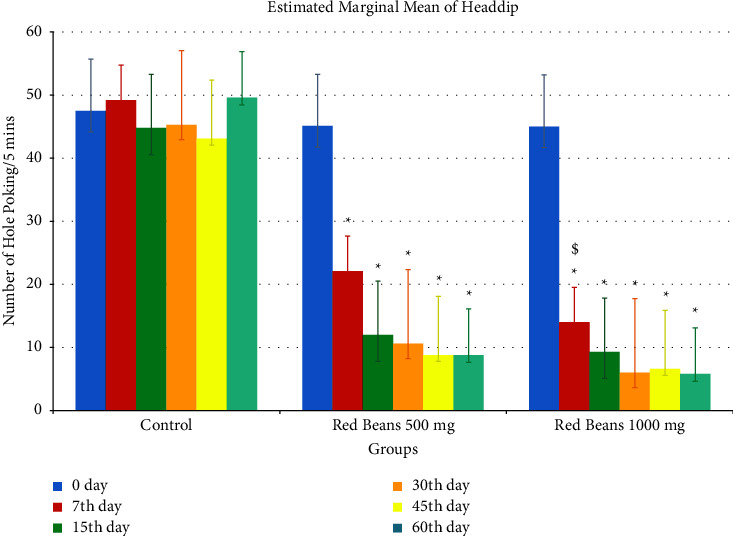
Head dip readings. ^*∗*^For control with other treated groups. ^$^For comparison with red bean 500 mg.

Figure 2 indicates the significance difference of control group with treated groups. Both the treated groups 500 mg/kg and 1000 mg/kg have remarkable anxiolytic effect.

#### 3.1.3. Open Field Center Box

**Figure 3:**
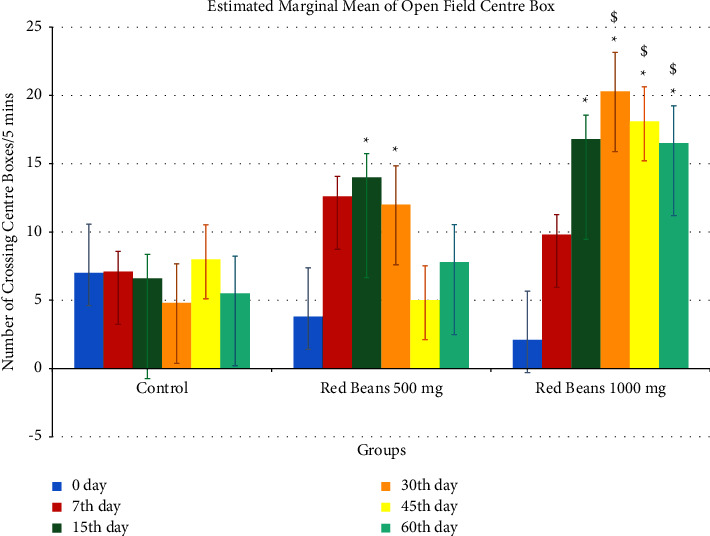
Open field center box readings. ^*∗*^For control with other treated groups. ^$^For comparison with red bean 500 mg.

Figure 3 shows the red bean effect on open field center box. The control group showed a significance difference with 500 mg/kg and 1000 mg/kg. It is also noticed that 500 mg/kg showed significance difference at 30th, 45th, and 60th day of 1000 mg/kg.

#### 3.1.4. Open Field Peripheral Box

**Figure 4:**
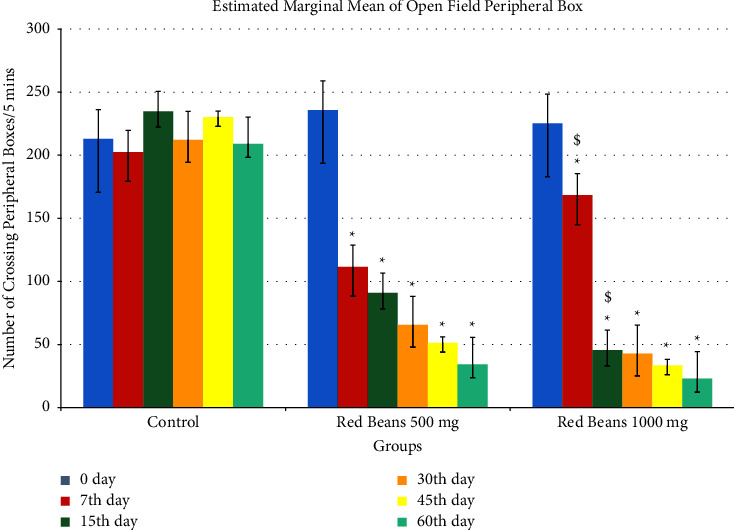
Open field peripheral box readings. ^*∗*^For control with other treated groups. ^$^For comparison with red bean 500 mg.

Figure 4 indicates anxiolytic effects as compared to control in both the treated groups. 500 mg/kg and 1000 mg/kg showed anxiolytic effect in overall days. On 7th and 15th day, 1000 mg/kg showed a significance difference as compared to 500 mg/kg.

#### 3.1.5. Elevated Plus Maze Open Arm

**Figure 5:**
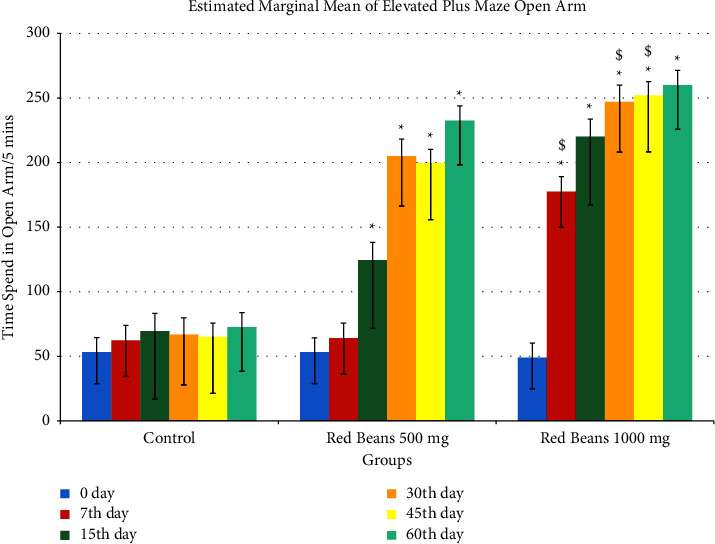
Elevated plus maze open arm readings. ^*∗*^For control with other treated groups. ^$^For comparison with red bean 500 mg.

Figure 5 shows the anxiolytic effect by spending more time in open arm. In comparison with the control group, both the treated groups showed a significance difference, but on 7th, 30th, and 45th day, 1000 mg/kg showed highly significant results as compared to 500 mg/kg.

#### 3.1.6. Light and Dark Test

**Figure 6:**
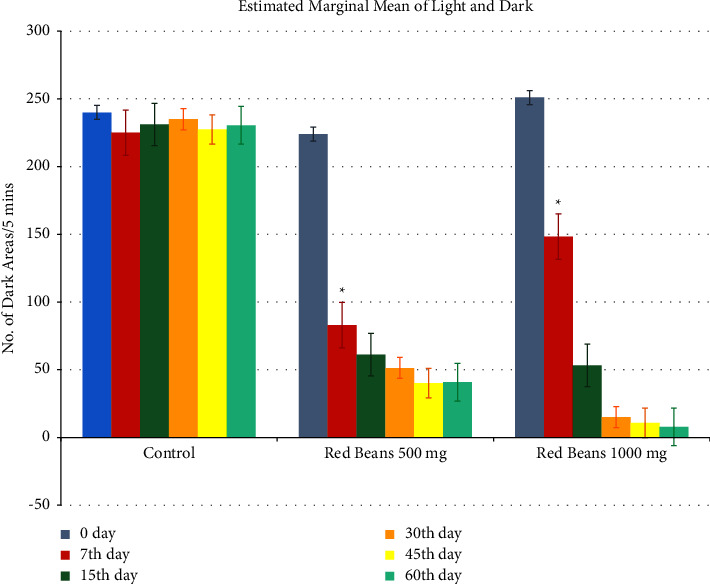
Light and dark readings. ^*∗*^For control with other treated groups. ^$^For comparison with red bean 500 mg.

Figure 6 shows the anxiolytic effect on light and dark apparatus. Control group showed a significance difference with comparison to both the treated groups. 1000 mg/kg showed more anxiolytic effect as compared to 500 mg/kg.

### 3.2. Depression Model

#### 3.2.1. Force Swimming Test

**Figure 7:**
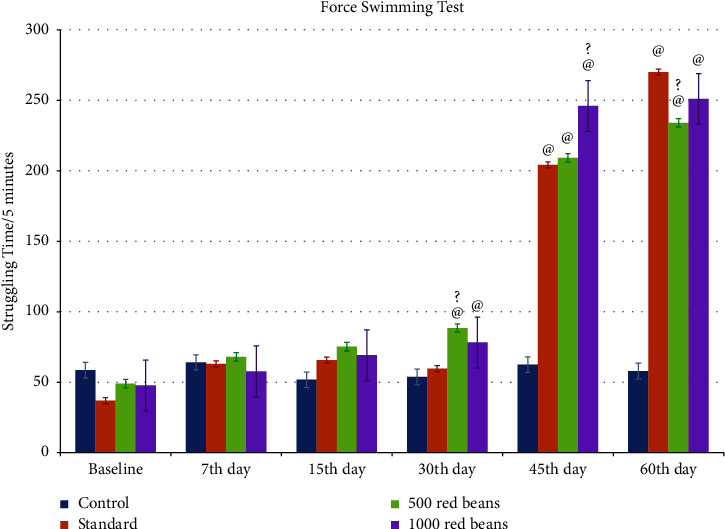
Results of force swimming test. ^@^Comparison from control. ^?^Comparison from standard.

Figure 7 shows the effect of *Phaseolus vulgaris* in different doses (500 mg/kg and 1000 mg/kg) on struggling time in forced swim test in rats.

Post hoc analysis by Tukey's test shows a highly significant (*p* ≤ 0.005) effect as compared to control throughout the treatment period by both doses of 500 mg/kg and 1000 mg/kg. Among the treated groups till day 30, there was no significant difference in the results; however, on the 45th day, 1000 mg/kg showed a significant increase in struggling time of rats as compared to standard.

#### 3.2.2. Corticosterone Levels

**Figure 8:**
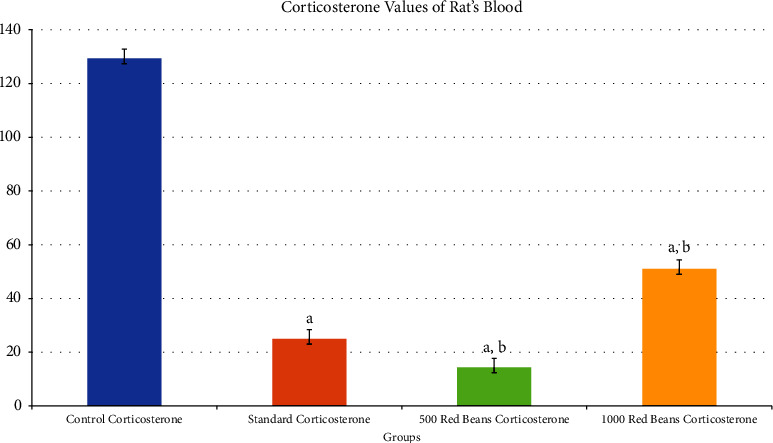
Corticosterone values of rat's blood. ^a^Comparison with control. ^b^Comparison with standard.

Figure 8 indicates that 1000 mg/kg dose showed a significant increase as compared to standard in corticosterone levels of treated rats.

The results of corticosterone analysis showed a significant decrease (*p* ≤ 0.005) in 500 mg/kg of red beans as compared to control and standard, whereas 1000 mg/kg showed a significant decrease as compared to control but not with the comparison of standard.

#### 3.2.3. Histopathological Evaluation of Rat Hippocampus

**Figure 9:**
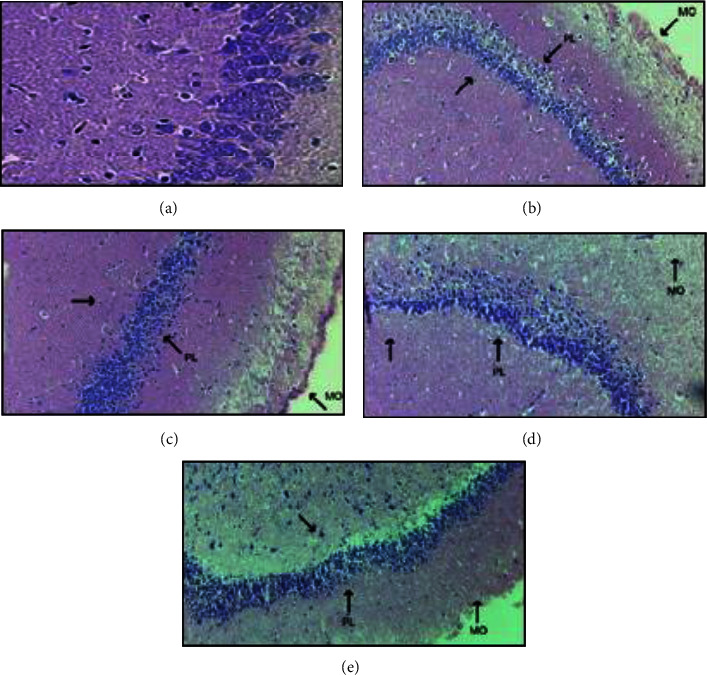
Histological slides of hippocampus. (a) Histological slide of hippocampus of depressed rat. (b) Sample 1 of 500 mg red beans. (c) Sample 2 of 500 mg red beans. (d) Sample 1 of 1000 mg red beans. (e) Sample 2 of 1000 mg red beans.

The histology of the hippocampus of a depressed rat is shown in Figure 9, where significant degenegeration of pyknotic nuclei in CA1 region is visible. It was also noted that the thickness of pyramidal layer in CA3 region of hippocampus was increased. The molecular layer and polymorphic layer became bigger in the depressed animal. Apoptotic cells were greater in the hippocampus of the depressed animal.

500 mg/kg *Phaseolus vulgaris* showed a significant increase in pyknotic nuclei. The pyramidal layer (PL) in the CA3 region had a focal reduction in thickness. The polymorphic layer (PO) and molecular layer (ML) have a focal reduction, and also, the apoptotic cells were reduced. 1000 mg/kg red beans indicated that the pyramidal layer in the CA3 region showed a focal reduction in thickness as compared to 500 mg/kg, and the molecular layer became thick. The polymorphic layer also became thin as compared to 500 mg/kg. After the treatment of 1000 mg/kg, the pykontic nuclei shrank and became pinpointed. They also showed a remarkable decrease in apoptotic cells.

#### 3.2.4. Gas Chromatography-Mass Spectroscopy Results for Erucic Acid and Optical Spectroscopy for Zinc Evaluation

**Figure 10:**
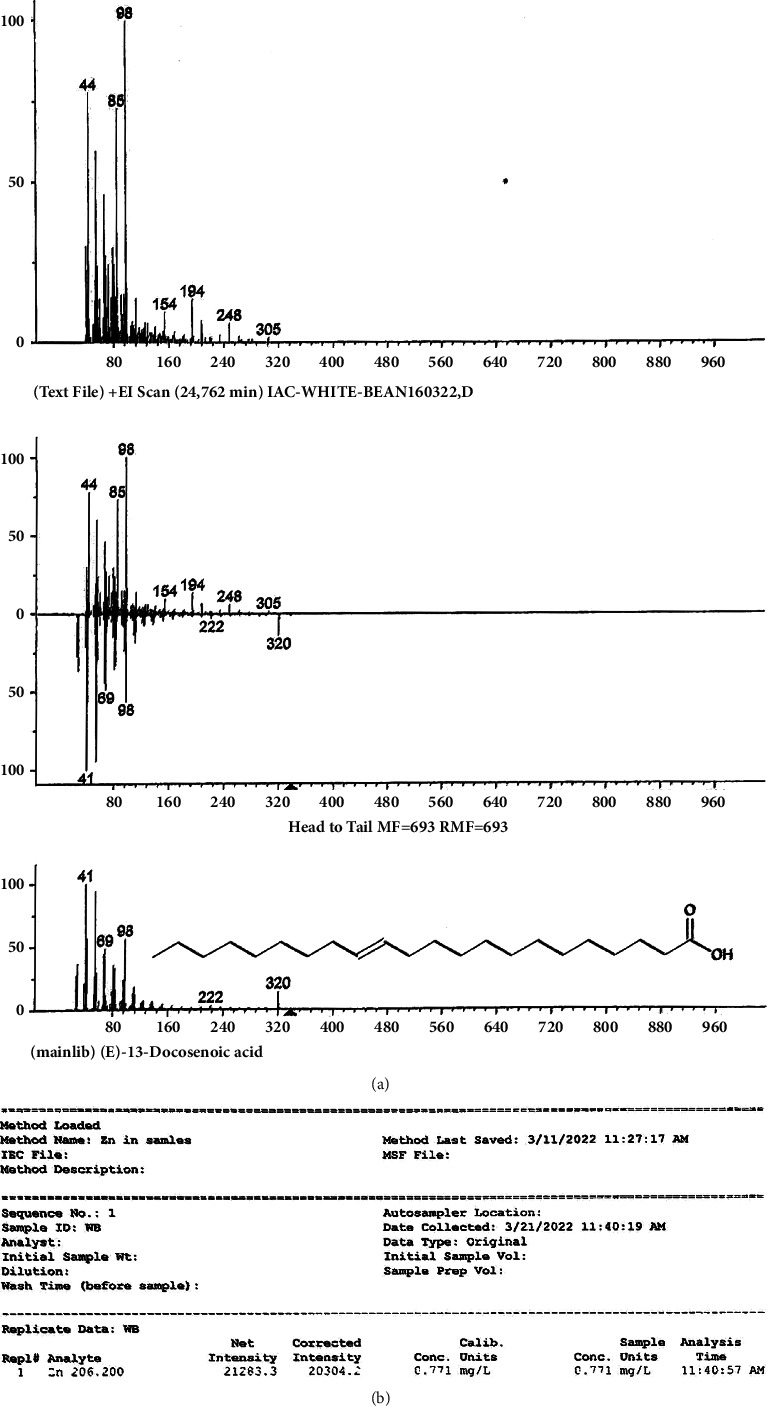
Evaluation of erucic acid and zinc in red beans. (a) Results of erucic acid peaks. (b) Concentration of zinc in red beans.

The peaks in Figure 10 show the presence of erucic acid also known as docosenoic acid at Retention Time 24.8.

The sample showed the presence of zinc which is about 2.306 mg/l.

## 4. Discussion

One of the most basic requirements of human life is food. It gives them the energy and nutrition to grow, develop, strengthen, and function efficiently. Nature has created many benefits in dietary products that can prevent and treat different diseases. Beans are considered an excellent contributor to micronutrients such as minerals and vitamins and are regarded to be superior to cereals in this aspect. Beans have the highest mineral concentration of any legume and are considered a significant source of iron, zinc, copper, aluminum, phosphorus, and other minerals in significant quantities. Red beans consist of many chemical compounds cited in references in Table 1 which are important for functional benefits of red beans.

The anxiety model results depict the anxiety decrease in all five apparatuses. As shown in Figure 2, the hole poke apparatus showed less poking from the 30th day to the 60th day. This is due to the presence of *α*-tocopherol, abundant in red beans [31]. *α*-Tocopherol helps improve the hypocellularity in the hippocampal dentate gyrus, ultimately reducing stress and anxiety [32]. Cage crossing and open field apparatus showed less anxiety on the 60th day, which showed that red beans have an anxiolytic effect due to proanthocyanidins [20] which increased the number of hippocampus neurons. However, it also reduces ROS production and blocks the activation of the NLRP3-Caspase-1 signaling pathway, thus reducing anxiety and stress, so it is recommended to incorporate kidney beans into a routine diet [33]. Multiple research studies have shown a possible connection between the estrous cycle and mood problems, namely, anxiety. Throughout various stages of the estrous cycle, there are changes in the levels of estrogen and progesterone. These fluctuations may affect neurotransmitter systems responsible for regulating mood, such as serotonin and gamma-aminobutyric acid (GABA). The activity of brain areas involved in emotional processing, such as the amygdala and prefrontal cortex, may be influenced by these hormone variations.

Studies conducted on animals have shown that variations in estrogen and progesterone levels during the estrous cycle may influence behaviors resembling anxiety. For instance, several studies have shown that rats display heightened anxiety-like behaviors during certain stages of the estrous cycle, characterized by elevated levels of estrogen and progesterone {Lovick, 2021 #319}.

The study's results in Figure 7 indicated a significant increase in the struggling time of rats in the force swimming test due to the presence of zinc in red beans [34]. The quantity of zinc present in *Phaseolus vulgaris* is 10.1 *μ*g/g [20]. The presence of zinc in our sample was indicated through optical analysis and is about 2.306 mg/l. When zinc attaches to its sensing receptors, which are GPR39, it activates the downstream cyclic MP-response element, dependent on gene transcription, resulting in high levels of brain-derived neurotrophic factor (BDNF) in specific brain regions, ultimately decreasing depression. If the zinc levels are low, it may cause high corticosterone production, resulting in hyperactivity of the hypothalamus-pituitary-adrenal (HPA) axis, which leads to depression. Corticosterone elevated levels reveal a link between zinc deficiency and depression [34].

The corticosterone levels in Figure 7 compared with depressed rats also revealed that treated rats with *Phaseolus vulgaris* have low levels of corticosterone; this is attributable to the presence of 13 decosenoic acids, also known as erucic acid, as mentioned through the process of GCMS. This is vital in reducing depression [20]. Erucic acid's primary function is to inhibit the enzyme *α*-amylase, a biomarker of depression and stress [35]. This inhibition of alpha-amylase reduces the HPA axis's functioning, resulting in decreased corticosterone levels [36].

Histological examination of the hippocampus in Figure 8 showed that the depressed rat hippocampus had significant degeneration of pyknotic nuclei in the CA 1 region, the thickness of the pyramidal layer in the CA3 region was also increased, and the apoptotic cells were higher in number in depressed rat while treated rat of 500 mg red beans showed shrinkage of pyknotic nuclei. In contrast, the pyramidal layer showed no change, but apoptotic cells were reduced compared to the disease model. 1000 mg/kg treated rats showed more remarkable changes in the hippocampus as pyknotic nuclei were significantly reduced, while the pyramidal layer also showed significant reduction, and apoptosis cells were nearly like those in the normal hippocampus. These changes in higher doses were due to flavonoids in *Phaseolus vulgaris*, which was 9.29 mg/1 gm. These flavonoids produce new-born cells that are progenitor cells in the hippocampus through neurogenesis's protective effect [37]. The *neurogenesis protective mechanism* is the process by which functional new neurons are generated from neural stem cells. This helps decrease many neuronal illnesses like stress, depression, and mood disorders [38].

The presence of melatonin shown in *Phaseolus vulgaris* also helps reduce depression in rats. Melatonin reduces apoptotic cells and degeneration of pyknotic nuclei by increasing monoamine oxidase levels in the hippocampus [39]. The quantity of melatonin in kidney beans is about 529.1 ng/g [40]. The overall results of our study indicated that kidney beans are very beneficial in treating depression because of the presence of many essential amino acids, flavonoids, minerals, and many other constituents.

## 5. Conclusion

A recent study concluded that anxiety and depression-related issues are certainly decreased with the help of natural products, especially red beans, which have many helpful constituents that reduce anxiety and depression, for example, proanthocyanidins, alpha tocopherols, zinc, erucic acid, melatonin, and flavonoids. The results of our study support the use of red beans in anxiety and depression.

## Figures and Tables

**Table 1:** Compounds found in red beans with their structure, molecular formula, and molecular weight.

Name of compound	Structure	Molecular formula	Molecular weight (g/mol)
Phaseolin	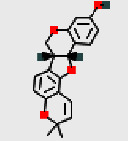	C_20_H_18_O_4_	322.4

*α*-Tocopherol	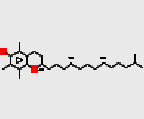	C_29_H_50_O_2_	430.7

Proanthocyanidins	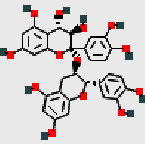	C_30_H_26_O_13_	594.5

Erucic acid	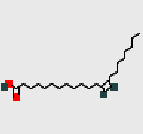	C_22_H_42_O_2_	338.6

Melatonin	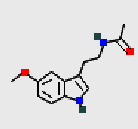	C_13_H_16_N_2_O_2_	232.2

## Data Availability

The datasets generated and/or analyzed during the current study are available from the corresponding author on reasonable request.

## Abbreviations

GCMS: Gas chromatography-mass spectroscopy
MDD: Major depressive disorder
SSRI: Selective serotonin reuptake inhibitors
GIT: Gastrointestinal tract
CCAC: Canadian Council on Animal Care
MO: Molecular layer
PL: Pyramidal layer
ICP-OES: Inductively coupled plasma-optical emission spectrometry
SIM: Selective ion monitoring.
